# Segmentation variability and radiomics stability for predicting triple-negative breast cancer subtype using magnetic resonance imaging

**DOI:** 10.1117/1.JMI.12.5.054501

**Published:** 2025-09-17

**Authors:** Isabella Cama, Alejandro Guzmán, Cristina Campi, Michele Piana, Karim Lekadir, Sara Garbarino, Oliver Díaz

**Affiliations:** a Università di Genova, Dipartimento di Matematica, Genova, Italy; b Università di Genova, Dipartimento di Neuroscienze, Riabilitazione, Oftalmologia, Genetica e Scienze Materno-Infantili, Genova, Italy; c Universitat de Barcelona, Departament de Matemàtiques i Informàtica, Barcelona, Spain; d IRCCS Ospedale Policlinico San Martino, Genova, Italy; e Institució Catalana de Recerca i Estudis Avançats (ICREA), Barcelona, Spain; f Universitat Autònoma de Barcelona, Computer Vision Center, Bellaterra (Cerdanyola del Vallès), Spain

**Keywords:** radiomics robustness, segmentation variability, triple-negative breast cancer subtype prediction, magnetic resonance imaging, explainability

## Abstract

**Purpose:**

Many studies caution against using radiomic features that are sensitive to contouring variability in predictive models for disease stratification. Consequently, metrics such as the intraclass correlation coefficient (ICC) are recommended to guide feature selection based on stability. However, the direct impact of segmentation variability on the performance of predictive models remains underexplored. We examine how segmentation variability affects both feature stability and predictive performance in the radiomics-based classification of triple-negative breast cancer (TNBC) using breast magnetic resonance imaging.

**Approach:**

We analyzed 244 images from the Duke dataset, introducing segmentation variability through controlled modifications of manual segmentations. For each segmentation mask, explainable radiomic features were selected using Shapley Additive exPlanations and used to train logistic regression models. Feature stability across segmentations was assessed via ICC, Pearson’s correlation, and reliability scores quantifying the relationship between segmentation variability and feature robustness.

**Results:**

Model performances in predicting TNBC do not exhibit a significant difference across varying segmentations. The most explicative and predictive features exhibit decreasing ICC as segmentation accuracy decreases. However, their predictive power remains intact due to low ICC combined with high Pearson’s correlation. No shared numerical relationship is found between feature stability and segmentation variability among the most predictive features.

**Conclusions:**

Moderate segmentation variability has a limited impact on model performance. Although incorporating peritumoral information may reduce feature reproducibility, it does not compromise predictive utility. Notably, feature stability is not a strict prerequisite for predictive relevance, highlighting that exclusive reliance on ICC or stability metrics for feature selection may inadvertently discard informative features.

## 1 Introduction

Breast cancer is a complex and heterogeneous disease, with multiple molecular subtypes that hamper accurate prediction of disease evolution and the development of targeted treatments.^1^

Triple-negative breast cancer (TNBC), one of the molecular subtypes, is defined by the absence of estrogen receptor (ER), progesterone receptor (PR), and human epidermal growth factor receptor 2 (HER2) overexpression, accounting for ∼15% to 20% of all breast cancers and disproportionately affecting younger and African American women.^2^^,^^3^ It is known to present a higher grade, earlier recurrence, and worse overall prognosis compared with other subtypes,^2^ thus representing the most challenging breast cancer subtype to treat.

Molecular subtype determination has always relied on techniques such as immunohistochemistry, staining, and fluorescence *in situ* hybridization.^1^ Recent advances in literature indicate the potential for predicting molecular subtypes using image-based features through machine learning (ML) techniques.^4^ These features, known as radiomic features, are quantitative descriptors extracted from medical images that capture detailed information about morphology, gray-level intensity, and texture within a specific region of interest (ROI), such as a tumor. The standard radiomics workflow requires an initial step of image segmentation,^5^^,^^6^ often relying on the annotator’s skill and the clarity of ROI boundaries.^7^ Then, radiomic features are extracted from the segmented images, and irrelevant or redundant features are discarded through feature selection procedures.^8^ Finally, predictive ML models are trained, and their application to diagnosis, prognosis, and treatment response prediction has been increasingly reported in the literature of breast cancer.^9^

Son et al.^10^ conducted a study on synthetic mammography, reconstructed from digital breast tomosynthesis, to predict the molecular subtype using both clinical and radiomic features. An elastic-net logistic regression^11^ model was trained to create the radiomics signature of each lesion. The results show that the combination of radiomics and clinical data outperformed the prediction using only clinical data, suggesting that radiomic signatures could serve as biomarkers for TNBC. In the case of magnetic resonance imaging (MRI), Leithner et al.^12^ combined radiomic data extracted from dynamic contrast-enhanced (DCE) MRI and apparent diffusion coefficient (ADC) map to differentiate TNBC from other subtypes. Overall, these studies showed that radiomics, i.e., imaging-based features, could support the identification of TNBC patients. By showing that imaging-based prediction can approximate biopsy-based classification of tumor subtype, the radiomics approach could potentially support earlier or supplementary decision-making in clinical workflows, offering a noninvasive way of analyzing tumor biology, especially in cases where biopsy may be risky, not feasible, or delayed.

However, the widespread adoption of radiomics in clinics is hampered by issues related to feature stability.^13^^,^^14^ Factors that could affect radiomic features computation can be found in the image acquisition and reconstruction phase, in the image preprocessing steps, and in the segmentation of the region of interest from which radiomic features are extracted. Scalco et al.^15^ reported a list of papers that evaluated the effects of segmentation on radiomics stability, with just one of them considering applications to breast cancer imaging. Granzier et al.^16^ studied the robustness of radiomic features, extracted by two software tools (RadiomiX and Pyradiomics^17^^,^^18^), with respect to variability in manual segmentation of breast tumors on MRI. Although a threshold value of 0.90 for the intraclass correlation coefficient (ICC)^19^ was chosen to determine feature robustness, its significance for radiomic models in predicting patient outcomes was not investigated.

More recently, Pistel et al.^20^ assessed the robustness of radiomics features in mammography by analyzing their sensitivity to ROI segmentation variations (underestimated, original, overestimated) for masses and calcifications across different views. Robustness was quantified using Kruskal–Wallis and pairwise tests, with higher p-values indicating more stable features. Findings show that features extracted from underestimated ROIs are more robust than those from overestimated ones, particularly for calcifications. The study focuses on feature stability rather than predictive accuracy, offering only qualitative insights into discriminative power. Ponsiglione et al.^21^ investigated the stability of radiomic features extracted from ADC maps of breast lesions under variations in segmentation mask shape and position. The results show that feature robustness varies depending on the type of segmentation alteration, in terms of overall concordance correlation coefficient and dynamic range. However, no predictive task is considered in this study.

Other studies addressed the problem of assessing the robustness of radiomic features by segmentation perturbation^22^^–^^24^ for various applications, various types of imaging [computed tomography (CT), positron emission tomography (PET), and MRI], and different diseases (lung cancer, head and neck squamous cell carcinoma, and glioblastoma). All of these studies call for caution in the use of predictive models involving radiomic features implicated in contour variability in the context of disease stratification and risk assessment, although no prediction experiments are reported.

Kothari et al.^25^ highlighted that the selection of robust features from masks delineated by different clinicians allows survival models to retain their prognostic ability. Among the papers addressing segmentation variability in a more systematic way, along with predictive tasks, Poirot et al.^26^ studied how differences among segmentations affect radiomic features in neuroimaging, in a cohort of T1-weighted and diffusion tensor MRI of sleep-deprived patients. The robustness and reproducibility of radiomic features were assessed using ICC for descriptive purposes, whereas the performance of the predictive model is only expressed in terms of accuracy, and no statistical analysis is reported to evaluate feature robustness in relation to predictive performance. Liu et al.^27^ showed that for CT images of oropharyngeal cancer, radiomic features varied a lot when the ROIs were not well segmented (ICC). No statistical test is reported for the significance of the results, and no quantification of the segmentation variability is presented. Moreover, the authors did not examine the behavior of the same group of features across variations in masks nor did they attempt to generate a new prognostic signature; instead, they trained univariate models for prediction. Similarly, Hatamikia et al.^28^ evaluated how small variations in tumor segmentation (±1 to 2 mm dilation/erosion, smoothing, ellipsoid approximation, and random distortions) affect MRI-based radiomic models predicting neoadjuvant chemotherapy (pCR) in 37 TNBC patients. Two analyses were performed, one using features selected from manual volumes of interest (VOIs) but tested on modified VOIs, and another with feature selection repeated for each modified VOI. The prediction results are reported on a single data split, without confidence intervals or standard deviations to quantify possible variability in the model predictive power. The model performance shows some sharp drops in terms of area under the receiver operating characteristic curve (ROC-AUC)—e.g., from 0.80 to 0.35 with slight dilation, and from 0.83 to 0.45 with slightly different contour randomization—raising concerns about sensitivity to even minor segmentation changes. The average ICC of selected features for each model is reported for descriptive purposes, showing no relationship with the goodness of the prediction result.

In summary, the existing literature used the ICC to evaluate radiomic features’ reproducibility as a synonym of stability, reliability, and robustness across different segmentation masks, and to perform feature selection. Of note, the ICC-based approach does not consider the possibility of significant quantitative differences in the various segmentation outcomes, implicitly assuming that the segmentation agreement is always high. Moreover, when analyzing feature stability exclusively with respect to segmentation, i.e., outside the context of prediction, specific features may exhibit a lack of robustness, which would be mostly mitigated during prediction due to the feature scaling required by ML methods. Only in a few cases is a prediction task performed on the selected robust features, usually on a fixed data split.

In this study, we investigate how segmentation variability impacts the stability of radiomic features and the performance of predictive models. We place particular emphasis on radiomics-based ML models to distinguish TNBC from other molecular subtypes using features extracted from breast MRI. First, we implement a comprehensive simulation strategy to produce over-segmentation, under-segmentation, and random alterations of manual lesion segmentations. Then, our analysis focuses on identifying radiomic features that consistently predict TNBC across the considered population of breast cancer patients. Furthermore, we explore the predictive performance and the stability of the most significant features across different segmentation masks. The stability of the features selected by the predictive models is evaluated using the ICC to enable comparison with existing literature, as well as Pearson’s correlation coefficient. In addition, we employ the method introduced in Ref. 14, which proposes four quantitative scores to assess feature stability in terms of consistency, robustness, instability, and the quality of feature computation. This method explicitly accounts for variability introduced during the segmentation process.

Although this study focuses on a specific clinical application, its findings may offer insights into the general behavior of radiomic features for predictive purposes, contributing to a broader understanding of the principles underlying radiomics-based analyses.

## 2 Materials and Methods

### 2.1 Data Collection

Consent or waiver for data usage was not required because all data were obtained in a de-identified form from the publicly available Duke-Breast-Cancer-MRI dataset,^29^ hosted on The Cancer Imaging Archive.^30^ For each patient, the Duke-Breast-Cancer-MRI dataset contains DCE-MR images from multiple time points (i.e., phases), capturing both pre-contrast and post-contrast phases. Imaging data were collected using various scanner models from GE MEDICAL SYSTEMS, MPTronic, and SIEMENS, including the Avanto, Optima MR450w, SIGNA EXCITE, SIGNA HDx, SIGNA HDxt, Skyra, Trio, and TrioTim. The dataset also contains demographic, clinical, pathology, and treatment information and outcomes of patients (e.g., response to treatment, recurrence, follow up). Pre-operative DCE-MR first-post-contrast images were used for this study. The patients involved correspond to the 251 selected by Caballo et al.^31^ Seven patients were excluded due to issues related to DICOM metadata. Therefore, a cohort of 244 patients was analyzed for this study, including 71 with TNBC and 173 non-TNBC (30% versus 70% of the dataset, respectively). Distributions of ER status, PR status, HER2 status, and molecular subtypes (HER2-enriched, Luminal A, Luminal B, and Triple Negative) are reported in Table 1.

**Table 1:** Distribution of ER status, PR status, HER2 status, and molecular subtypes in the used dataset.

Category	Label	Frequency	Percentage (%)
ER status	Negative	100	41.00
	Positive	144	59.00
PR status	Negative	132	54.10
	Positive	112	45.90
HER2 status	Negative	176	72.10
	Positive	68	27.90
Molecular subtype	HER2-enriched	23	9.40
	Luminal A	105	43.00
	Luminal B	45	18.40
	Triple negative	71	29.10

### 2.2 Segmentation

Manual breast lesion segmentation at the MR images was provided by Caballo et al.^31^ To assess the stability of the features with respect to segmentation accuracy, we introduced variability in the manual segmentation mask by simulating seven other annotations per case. Our aim was to obtain an average mean dice similarity coefficient (DSC) across all segmentations ranging from 0.4 to 0.8 with respect to the original mask. This was achieved via the morphological operations of closing and opening. Using the ball structuring element from the skimage.morphology library^32^ with varying kernel sizes, we gradually enlarged (closing) and reduced (opening) the manual segmentation mask to achieve various mean DSC, including more and more portions of the peritumoral region (closing) or excluding part of the tumoral tissue (opening). Specifically, we applied the following operations.

For the closing modifications (over-segmentation):

• dilation and erosion operations, in this order, using a kernel size of 5, to obtain a mean DSC of 0.8;
• dilation and erosion, using a kernel size of 9, to obtain a mean DSC of 0.7;
• dilation with a kernel size of 11 and erosion with a kernel size of 9, to obtain a mean DSC of 0.6;
• finally, the manual segmentation was replaced by the ellipsoid contained in the ROI box provided by the dataset, along with the images, to obtain a mean DSC of 0.4.

For the opening modifications (under-segmentation):

• erosion and dilation operations, in this order, with a kernel size of 5 for both operations, to obtain a mean DSC of 0.8;
• erosion and dilation, with a kernel size of 9 for both operations, to obtain a mean DSC of 0.7;
• erosion and dilation, using a kernel size of 11 for both operations, to obtain a mean DSC of 0.6.

**Fig. 1:**
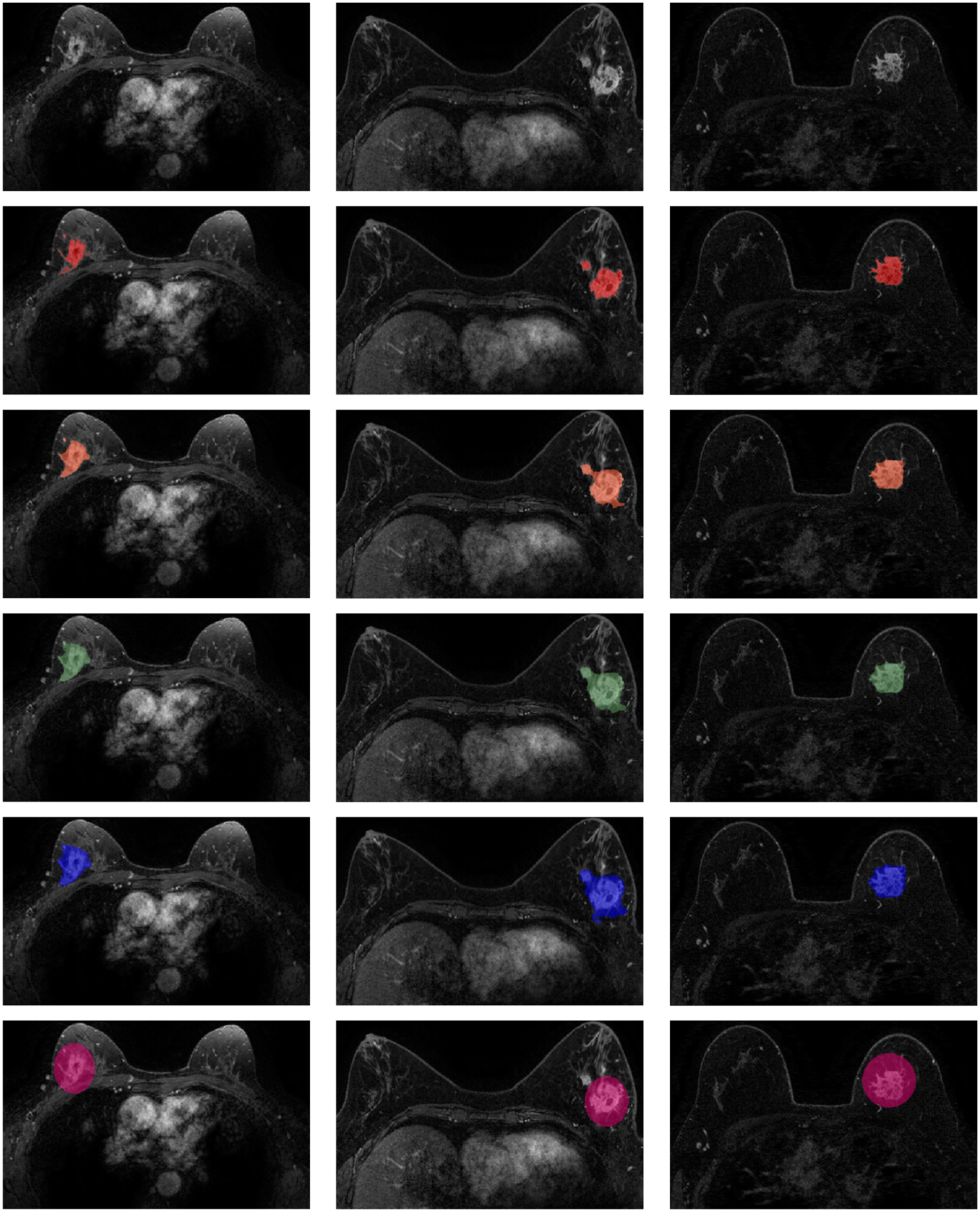
Left to right: MR slices of three different patients (first-post-contrast image). Top to bottom: original image, manual tumor segmentation (red), closing 08 mask (orange), closing 07 (green), closing 06 (blue), and ellipsoid 04 (magenta). For the definition of “closing mask,” see Sec. 2.2.

**Fig. 2:**
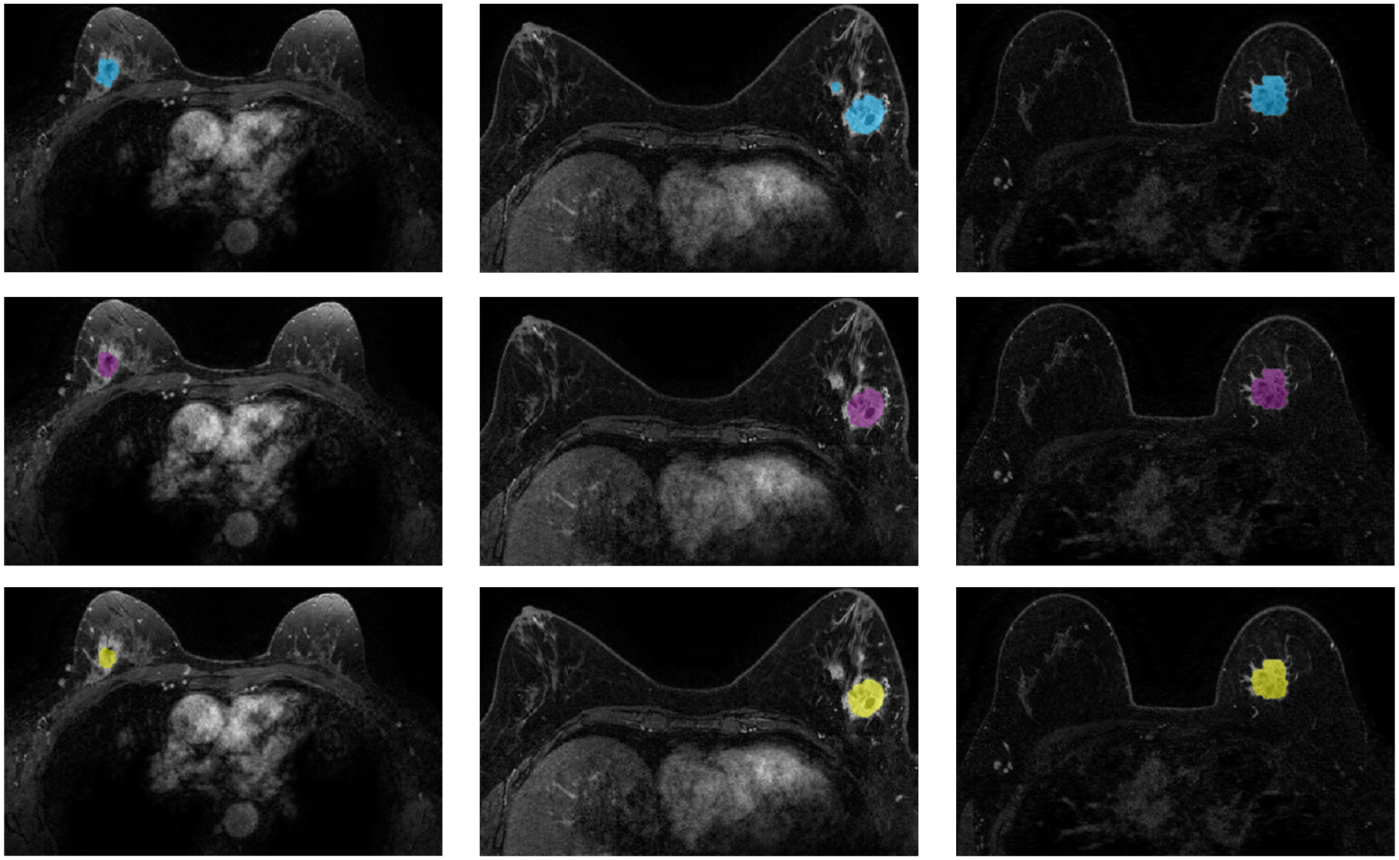
Left to right: MR slices of three different patients (first-post-contrast image). Top to bottom: opening 08 mask (cyan), opening 07 (purple), and opening 06 (yellow). For the definition of “opening mask,” see Sec. 2.2.

The implemented modifications of the manual masks allowed for consideration of a broad range of potential contour variations as the segmentation obtained through morphological operations exhibited significant variability. For instance:

• certain tumors were originally located near the skin, causing the modified mask to extend beyond the patient’s body;
• some segmentations were shrunk to the tumor core, excluding part or most of the tumoral tissue (Fig. 2, left column);
• segmentation of tumors with multiple lesions resulted in being grouped into a single mask (Fig. 1, central column), or one lesion was excluded from segmentation (Fig. 2, central column);
• spike-like structures were enhanced (Fig. 1, in orange).

In addition, the ellipsoid mask served as a geometric approximation of tumor segmentation (DSC 0.4), fulfilling the same function as a ROI box by providing a highly simplified approximation while offering a more natural shape, closer to a potential manual segmentation.

In the rest of the document, the manual mask and its modifications are referred to as manual, closing 08, closing 07, closing 06, ellipsoid 04, opening 08, opening 07, and opening 06, respectively (see Figs. 1 and 2), referring to the mean DSC value reached by each morphological operation applied to the original mask. To address the heterogeneity and stochastic variability among human observers, three additional segmentation sets were created by randomly sampling, for each patient, one segmentation mask among (i) manual segmentation, closing, and ellipsoid masks; (ii) manual segmentation and opening masks; and (iii) all the proposed segmentation masks together. Introducing randomly assigned segmentation modifications within the cohort better simulates realistic segmentation variability and improves the robustness of the evaluation conducted in this study. These segmentations are referred to as “random manual-closing-ellipsoid,” “random manual-opening,” and “random manual-closing-ellipsoid-opening,” respectively.

### 2.3 Radiomic Feature Extraction

Radiomic features were extracted from the segmentation masks after image *z*-score normalization and fixed-bin count discretization with 50 bins using the PyRadiomics 3.1.0 library.^18^ We extracted 1130 radiomic features, including shape, texture, matrix-based, wavelet, and Laplacian of Gaussian (LoG) features (σ=1, 2, 3).

Shape-based features describe the geometric properties of a region of interest, such as volume, surface area, and compactness. We observe that shape features are inherently less meaningful in our context given that we apply deliberate geometric variations to the segmentations. Nonetheless, for completeness and consistency with international standard radiomics workflows,^6^ we extracted all shape features using a conventional pipeline. First-order statistics capture the distribution of voxel intensities within the image, summarizing global intensity characteristics. Gray-level co-occurrence matrix (GLCM) features assess spatial relationships among intensity values, reflecting textural uniformity and contrast. Gray-level run-length matrix (GLRLM) features quantify the length and distribution of consecutive voxel intensity runs, characterizing texture smoothness. Gray-level size zone matrix (GLSZM) features evaluate the size and distribution of homogeneous intensity zones. Neighboring gray-tone difference matrix (NGTDM) features measure texture strength and contrast based on intensity differences with neighboring voxels. Gray-level dependence matrix (GLDM) features capture the degree to which voxels depend on neighboring intensities, quantifying image granularity and texture complexity.

All these features were extracted from the original and filtered images. Wavelet-HHH, wavelet-HLH, and wavelet-LLL filters are types of wavelet transform filters^33^ where HHH applies high-pass filtering across all three dimensions to capture high-frequency features, HLH applies a combination of high- and low-pass filtering in different dimensions to extract mixed frequency information, and LLL applies low-pass filtering across all dimensions to capture the overall low-frequency components. On the other hand, LoG^34^ is an edge detection method where σ values denote the scale of Gaussian smoothing applied before computing the Laplacian to detect edges at varying levels of detail. Extensive documentation of radiomics features can be found in Ref. 18.

For the analysis, features were normalized using z-score. Then, they were harmonized to limit the potential bias introduced by the differences in signal-to-noise ratio caused by the manufacturer model. Harmonization was performed using the parametric version of the ComBat method,^35^^,^^36^ which yields a transformation of the feature distributions according to the variable being tested (manufacturer model) using additive and multiplicative batch effects.

### 2.4 Clinical Features

Clinically derived features were utilized for prediction purposes to enable comparisons with radiomics-based models. Specifically, demographical variables (i.e., age, menopause at diagnosis, ethnicity, and metastatic state at presentation) and biopsy variables (i.e., tubule formation, nuclear grade, and mitotic rate) were considered. These biopsy variables are clinically used for breast cancer grading and are strongly associated with tumor aggressiveness. Although they do not directly determine tumor molecular subtype, they may be naturally linked to it as higher histologic grades often correlate with more aggressive subtypes. For this reason, the model based on biopsy variables could serve as a reference for comparison in subtype prediction.

### 2.5 Machine Learning Models

The dataset was initially split into stratified training and testing subsets using a 70% to 30% ratio, repeated across 130 random data splits to evaluate the performance of the ML models. The number of data splits was chosen heuristically as it corresponds to approximately half the size of our dataset (244 patients). Specifically, we aimed to generate a large number of splits to ensure a broad representation of feature selection outcomes across data splits, even in cases where only a few patients differed between splits.

For each data split, the imbalanced data issue (TNBC versus non-TNBC) was addressed prior to the training process via the synthetic minority oversampling technique^37^ (SMOTE), a widely used method for random oversampling of tabular data, such as radiomic features. A preliminary selection of the 50 most informative features was performed through ANOVA F-values computation, used to rank features based on their relevance to the target variable. Then, a logistic regression model with L1-norm penalty^38^ was trained for the classification task TNBC versus non-TNBC with fivefold cross-validation. The regularization parameter C, to control feature selection strength, was set to 1. L1-norm penalty is known to promote sparsity by selecting only the most relevant features for the model, thus identifying the radiomic signature of each lesion.

**Fig. 3:**
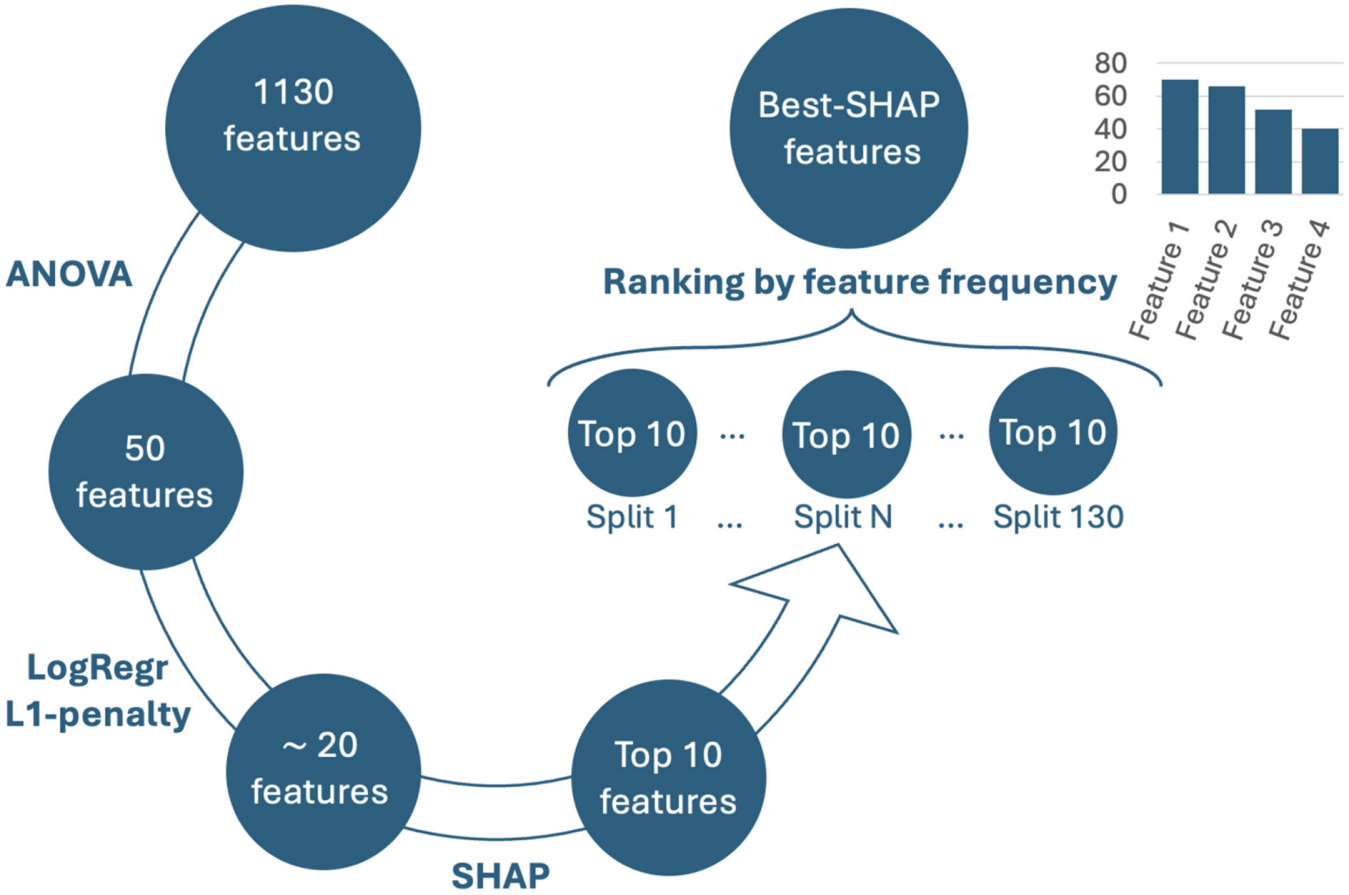
Flowchart of the feature selection methodology employed for this study, based on the SHAP explainability algorithm.

After the training process, we used the SHAP algorithm (Shapley Additive exPlanations^39^) to collect the most explicative features for each model (each trained on a different data split). SHAP is a feature importance tool, based on a game theoretic approach, used in ML for explaining the output of a model by quantifying the importance of each feature. SHAP identifies the most relevant features that contribute to the model’s predictions by calculating SHAP values for each feature in the dataset: features with higher SHAP values are considered more influential in the model’s predictions, whereas features with lower SHAP values have less impact. Specifically, for each trained model, we identified the top 10 features selected by SHAP. From the aggregated set of top SHAP-selected features across all models, we further selected those with the highest frequency of occurrence that appeared at least 15 times overall (the “best-SHAP features” from now on). These features can be considered collectively predictive as they contributed to the predictive performance across the whole dataset. A diagram outlining the SHAP feature selection methodology employed in this study is shown in Fig. 3, whereas a detailed implementation is reported in Algorithm 1. The best-SHAP features were then used to train logistic regression models, one for each data split, for comparison with the baseline models, described at the beginning of this section. This procedure was repeated for each feature set, extracted from the segmentation masks described in Sec. 2.2. In the following, “baseline-manual,” “baseline-closing 08,” “baseline-closing 07,” “baseline-closing 06,” “baseline-ellipsoid 04,” “baseline-opening 08,” “baseline-opening 07,” “baseline-opening 06,” “baseline-random manual-closing-ellipsoid,” “baseline-random manual-opening,” and “baseline-random manual-closing-ellipsoid-opening” will refer to baseline models trained with features extracted from the corresponding mask, whereas “best-SHAP-manual,” “best-SHAP-closing 08,” “best-SHAP-closing 07,” “best-SHAP-closing 06,” “best-SHAP-ellipsoid 04,” “best-SHAP-opening 08,” “best-SHAP-opening 07,” “best-SHAP-opening 06,” “best-SHAP-random manual-closing-ellipsoid,” “best-SHAP-random manual-opening,” and “best-SHAP-random manual-closing-ellipsoid-opening” will indicate models trained with best-SHAP features derived from the corresponding baseline model. All ML models were developed in Python (v3.12.2).

**Algorithm 1:** Best-SHAP feature selection.

Input: radiomic features extracted from the manual segmentation
**Step 1: Preliminary feature selection and model training**
For each data split (training set):
1. compute ANOVA F-values to rank features based on their relevance to the target (TNBC)
2. select the first 50 features
3. train the predictive model with such features (each LogReg model further selects ∼20 features due to L1 penalty)
4. collect the top 10 features considered explainable for the current model by SHAP method
**Step 2: best-SHAP feature selection**
From the aggregated set of SHAP-selected features identified in step 1:
1. count the frequency of occurrence of each feature
2. rank them in descending order
3. select the ones that appeared at least 15 times overall: these are the best-SHAP features for the manual segmentation
Repeat steps 1 and 2 for each feature set (closing 08, 07, 06, ellipsoid 04, opening 08, 07, 06, random manual-closing-ellipsoid, random manual-opening, random manual-closing-ellipsoid-opening).

### 2.6 Statistical Analysis

Univariate statistical analysis was performed to evaluate the potential significance of individual descriptors in discriminating TNBC versus non-TNBC cases. Continuous variables were analyzed with two-sided Kolmogorov–Smirnov (KS) test, whereas discrete variables were analyzed with Pearson’s chi-squared test. Bonferroni correction for multiple comparisons was applied.

Prediction models, leveraging clinical, radiomic, and the best-SHAP features, underwent rigorous evaluation for various performance metrics: accuracy, recall, specificity, balanced accuracy (which accounts for dataset imbalance by computing the mean of recall and specificity), and ROC-AUC. The performances reported in this paper are averaged across data splits, and 95% confidence intervals (CI) are reported. The Kolmogorov–Smirnov test was used to evaluate the difference in score distributions from different ML models, with Bonferroni correction for multiple comparisons.

To check feature stability/robustness across different segmentation masks, the most relevant features for the models were evaluated via ICC^19^ and Pearson’s correlation coefficient. The ICC measures agreement between measurements, and in radiomics, is often used to assess feature reproducibility across different segmentation masks (representing the raters). This study utilized a two-way random effects model, generally denoted as ICC(2), which is appropriate when all raters evaluate all subjects and both raters and subjects are considered random effects. The computation accounts for variability between subjects and raters, as well as error variance, which may result from differences in rater performance or other unaccounted noise. ICC and Pearson’s correlation were computed pairwise using the features extracted from the manual mask and each of its modifications.

To evaluate feature stability, we also used the scores proposed by Cama et al.,^14^ measuring the numerical relationship between feature stability and tumor segmentation. These “reliability” scores are based on a quantitative assessment of segmentation variability and the relative error on feature values, where “reliability” refers to feature stability with respect to segmentation variability:

• the quality score indicates when segmentation agreement and feature computation accuracy are simultaneously high (see purple quality area in Fig. 9);
• the consistency score highlights anti-linear correlation of feature error and segmentation result (the error grows while the DSC decreases, see light blue consistency area in Fig. 9);
• the robustness score indicates independence of feature value from segmentation variability (see green robustness area in Fig. 9);
• the instability score shows high dependence of feature value on minor segmentation variations (see light red instability area in Fig. 9).

These scores can serve as quantitative parameters for a reliability/stability assessment process in radiomics. Specifically, for each feature, the scores provide measures of the collective behavior of that feature on a group of patients, by quantifying the proportion of patients with a specific trend of feature stability. However, their interpretation is tied to the distribution of the DSC along the x-axis. In this study, the DSC was deliberately adjusted to achieve specific mean values, concentrating the analyses on specific portions of the x-axis for each case.

## 3 Results

Table 2 displays the means and standard deviations of the DSC obtained for the various modifications of the manual segmentation mask, computed across the patients involved in the study.

**Table 2:** Mean ± standard deviation of the DSC obtained for each modification of the manual segmentation mask, computed across all the patients.

Segmentation mask	Mean DSC ± standard deviation
Closing 08	0.81 ± 0.13
Closing 07	0.69 ± 0.12
Closing 06	0.60 ± 0.12
Ellipsoid 04	0.40 ± 0.13
Opening 08	0.76 ± 0.17
Opening 07	0.68 ± 0.20
Opening 06	0.62 ± 0.23

The univariate feature analysis, performed on the whole dataset, showed that no single feature independently exhibited a significant difference between the TNBC and non-TNBC groups based on the Kolmogorov–Smirnov and Chi-squared tests (p-value>0.05).

**Fig. 4:**
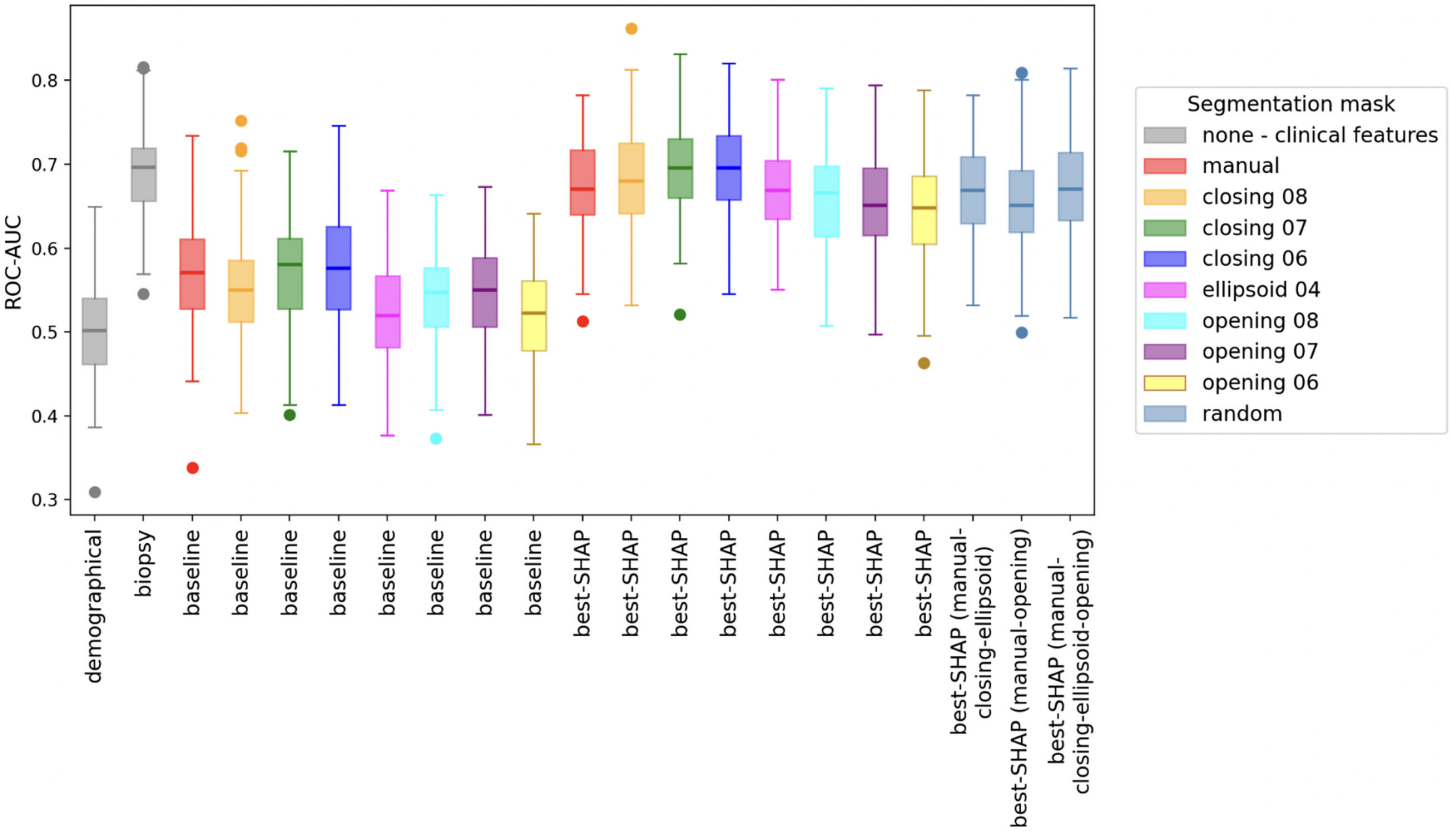
ROC-AUC scores obtained by testing demographical model (boxplot 1), biopsy model (boxplot 2), baseline models (boxplots 3 to 10), and best-SHAP models (boxplots 11 to 21). Due to space reasons, baseline random models are not reported in this figure.

Figure 4 illustrates the performance of the prediction models using demographical variables (boxplot 1) and biopsy variables (boxplot 2), as well as baseline models (boxplots 3 to 10) and best-SHAP models (boxplots 11 to 21). Performance is shown in terms of ROC-AUC. Table 3 reports detailed statistics, including skill-score means and confidence intervals obtained for each ML model. The prediction using solely demographical variables is random.

**Table 3:** Performances of demographical model, biopsy model, baseline models, and best-SHAP models for TNBC prediction. Mean skill-scores and their 95% confidence interval (within brackets) are reported. Bold denotes the best mean result for each skill-score.

Experiment	Accuracy	Balanced accuracy	Recall	Specificity	ROC-AUC
Demographical	0.482 (0.473, 0.490)	0.492 (0.483, 0.501)	0.517 (0.497, 0.537)	0.467 (0.454, 0.480)	0.499 (0.488, 0.509)
Biopsy	0.607 (0.598, 0.615)	**0.651** (0.642, 0.661)	**0.760** (0.739, 0.782)	0.543 (0.528, 0.557)	0.687 (0.677, 0.697)
Baseline-manual	0.588 (0.579, 0.596)	0.553 (0.543, 0.563)	0.468 (0.447, 0.488)	0.638 (0.628, 0.649)	0.575 (0.563, 0.586)
Baseline-closing 08	0.567 (0.558, 0.576)	0.532 (0.523, 0.542)	0.446 (0.428, 0.464)	0.619 (0.607, 0.631)	0.552 (0.542, 0.563)
Baseline-closing 07	0.591 (0.581, 0.600)	0.553 (0.543, 0.564)	0.462 (0.443, 0.481)	0.645 (0.632, 0.658)	0.573 (0.562, 0.584)
Baseline-closing 06	0.591 (0.581, 0.601)	0.555 (0.544, 0.566)	0.468 (0.448, 0.488)	0.643 (0.629, 0.656)	0.578 (0.566, 0.590)
Baseline-ellipsoid 04	0.560 (0.551, 0.569)	0.516 (0.506, 0.526)	0.407 (0.390, 0.425)	0.625 (0.612, 0.637)	0.524 (0.514, 0.535)
Baseline-opening 08	0.571 (0.562, 0.579)	0.533 (0.524, 0.541)	0.439 (0.422, 0.456)	0.626 (0.614, 0.639)	0.538 (0.528, 0.548)
Baseline-opening 07	0.578 (0.570, 0.586)	0.543 (0.534, 0.552)	0.457 (0.439, 0.476)	0.629 (0.618, 0.641)	0.547 (0.536, 0.557)
Baseline-opening 06	0.558 (0.550, 0.566)	0.517 (0.508, 0.526)	0.415 (0.397, 0.433)	0.619 (0.607, 0.630)	0.521 (0.511, 0.532)
Baseline-random manual-closing-ellipsoid	0.584 (0.575, 0.594)	0.550 (0.540, 0.559)	0.463 (0.444, 0.483)	0.636 (0.623, 0.649)	0.566 (0.555, 0.577)
Baseline-random manual-opening	0.578 (0.568, 0.589)	0.539 (0.529, 0.550)	0.443 (0.425, 0.460)	0.636 (0.622, 0.650)	0.541 (0.530, 0.553)
Baseline-random manual-closing-ellipsoid-opening	0.587 (0.577, 0.597)	0.544 (0.533, 0.555)	0.445 (0.426, 0.464)	0.643 (0.630, 0.655)	0.548 (0.536, 0.560)
Best-SHAP-manual	0.643 (0.635, 0.651)	0.624 (0.615, 0.633)	0.577 (0.560, 0.595)	0.671 (0.660, 0.683)	0.673 (0.664, 0.683)
Best-SHAP-closing 08	0.647 (0.638, 0.655)	0.633 (0.624, 0.642)	0.598 (0.582, 0.615)	0.667 (0.655, 0.679)	0.685 (0.675, 0.695)
Best-SHAP-closing 07	0.655 (0.648, 0.663)	0.638 (0.629, 0.647)	0.595 (0.575, 0.615)	0.681 (0.670, 0.692)	0.695 (0.686, 0.705)
Best-SHAP-closing 06	**0.671** (0.663, 0.678)	0.649 (0.641, 0.657)	0.595 (0.577, 0.614)	**0.703** (0.690, 0.715)	**0.696** (0.686, 0.706)
Best-SHAP-ellipsoid 04	0.639 (0.632, 0.647)	0.618 (0.610, 0.627)	0.566 (0.549, 0.583)	0.671 (0.660, 0.681)	0.671 (0.662, 0.680)
Best-SHAP-opening 08	0.627 (0.618, 0.637)	0.609 (0.599, 0.619)	0.564 (0.546, 0.582)	0.654 (0.642, 0.667)	0.659 (0.648, 0.670)
Best-SHAP-opening 07	0.629 (0.620, 0.638)	0.607 (0.598, 0.616)	0.553 (0.536, 0.571)	0.661 (0.648, 0.674)	0.652 (0.641, 0.663)
Best-SHAP-opening 06	0.617 (0.609, 0.625)	0.598 (0.589, 0.607)	0.552 (0.534, 0.569)	0.644 (0.633, 0.655)	0.645 (0.634, 0.656)
Best-SHAP-random manual-closing-ellipsoid	0.640 (0.632, 0.649)	0.634 (0.625, 0.643)	0.620 (0.602, 0.637)	0.649 (0.637, 0.660)	0.670 (0.661, 0.679)
Best-SHAP-random manual-opening	0.653 (0.644, 0.661)	0.623 (0.614, 0.631)	0.548 (0.531, 0.565)	0.697 (0.685, 0.709)	0.655 (0.645, 0.665)
Best-SHAP-random manual-closing-ellipsoid-opening	0.662 (0.654, 0.670)	0.637 (0.627, 0.647)	0.579 (0.560, 0.598)	0.695 (0.684, 0.705)	0.667 (0.656, 0.678)

Kolmogorov–Smirnov tests provided evidence against equal model performance for all the comparisons between baseline models and best-SHAP models (for visual reference, see Fig. 4). No statistical significance is observed between the performances of the biopsy-based model and best-SHAP models, except for best-SHAP opening 07, best-SHAP opening 06, and best-SHAP random manual-opening models. Likewise, pairwise comparisons among the best-SHAP models across all masks detected only a few differences in model performance, highlighted in Table 4. Most of the significant differences were found between the closing and opening models. Table 4 provides a summary of these key comparisons.

**Table 4:** Results of the Kolmogorov–Smirnov tests to evaluate differences in model performance. Bonferroni correction for multiple comparisons was applied. Significance column: “*” indicates statistical evidence against equal model performance after Bonferroni correction; “—” means no statistical significance.

Model 1	Model 2	Significance
Baseline model (any mask)	Best-SHAP model (any mask)	*
Biopsy model	Best-SHAP model (any mask)	—^a^
Best-SHAP closing 08, 07, 06, ellipsoid 04	Best-SHAP closing 08, 07, 06, ellipsoid 04	—^b^
Best-SHAP opening 08, 07, 06	Best-SHAP opening 08, 07, 06	—
Best-SHAP closing 08	Best-SHAP opening 06	*
Best-SHAP closing 07, 06	Best-SHAP opening 08, 07, 06, and Best-SHAP random manual-opening	*

a Exception: statistical significance between biopsy and best-SHAP opening 07, 06, and best-SHAP random manual-opening.

b Exception: statistical significance between best-SHAP-closing 07 and best-SHAP-ellipsoid 04.

**Fig. 5:**
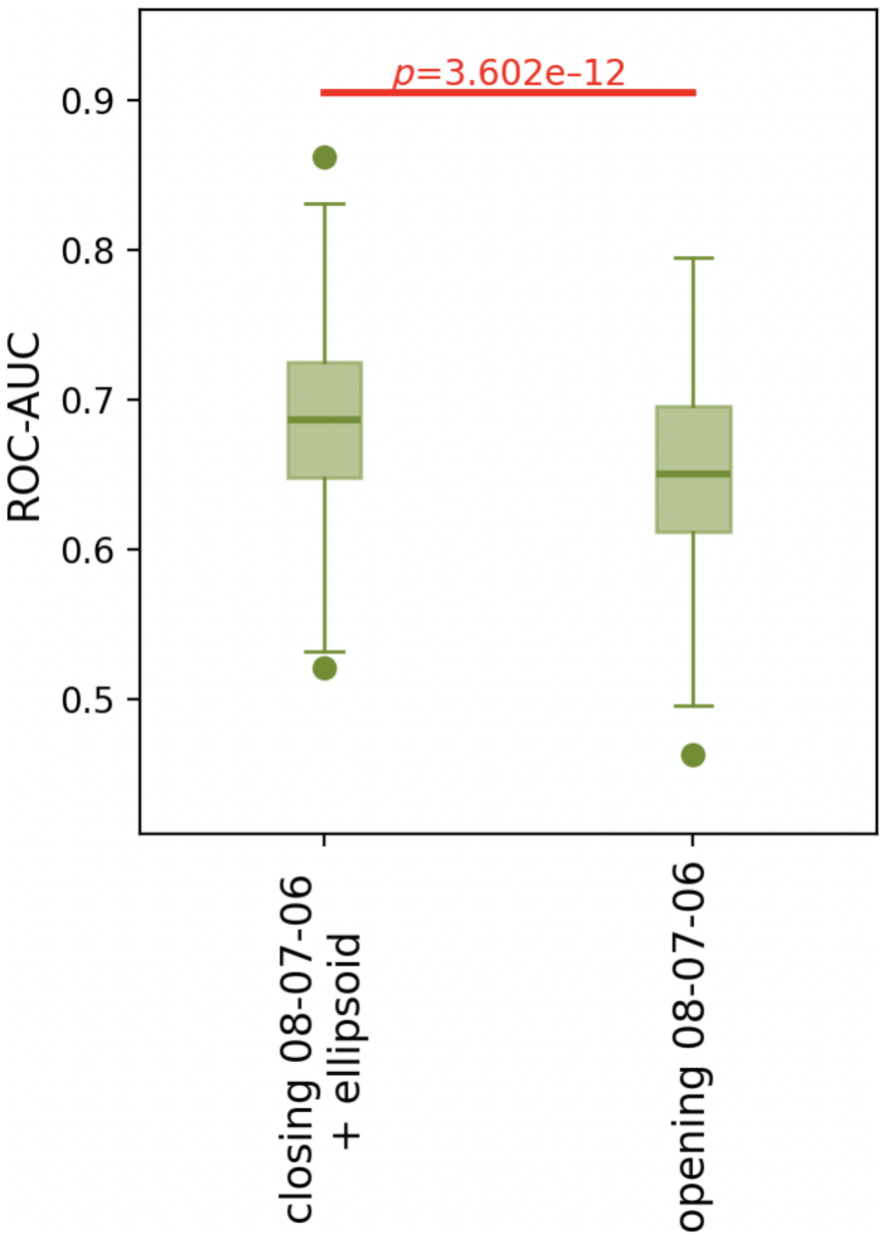
ROC-AUC scores obtained by aggregating the performances of the best-SHAP closing models (08, 07, 06, and ellipsoid) versus the best-SHAP opening models (08, 07, 06). Statistical difference shown with a red bar and p-value.

Figure 5 shows a comparison between the aggregated performances of the best-SHAP closing models (08, 07, 06, and ellipsoid) versus the best-SHAP opening models (08, 07, 06), indicating a p-value<0.05, i.e., a significant difference between the two score distributions.

Given the overall better performances of the closing-ellipsoid models against the opening models (see Fig. 5), we concentrated the analyses on feature stability on the best-SHAP manual, closing 08, 07, 06, and ellipsoid models (over-segmentation models). Table 5 lists the best-SHAP-features for each of these models, identified as the most overall explicative features for each segmentation mask, as described in Sec. 2.5. None of the shape features were selected by any of the models during the feature selection process, not even in the model based on features extracted from the manual mask. This outcome reinforces the limited stability and interpretability of shape features under our experimental conditions.

**Table 5:** List of the best-SHAP features identified as explicative through the SHAP method for each model (manual and over-segmentation models). “X” indicates the model for which the feature has been selected as explicative. Features that are selected by all the models are highlighted in bold.

Image filter	Matrix	Feature name	Manual	Closing 08	Closing 07	Closing 06	Ellipsoid 04
LoG σ=2	glcm	Cluster prominence	X	—	—	—	—
LoG σ=3	glrlm	Run entropy	X	X	—	—	—
**LoG** σ=3	**glszm**	**High-gray-level-zone emphasis**	**X**	**X**	**X**	**X**	**X**
LoG σ=3	glszm	Low-gray-level-zone emphasis	—	—	—	X	—
LoG σ=3	glszm	Small-area-high-gray-level emphasis	X	X	—	—	—
Original	firstorder	Median	—	—	X	—	—
Original	firstorder	Minimum	—	X	X	X	X
**Original**	**gldm**	**Large-dependence-high-gray-level emphasis**	**X**	**X**	**X**	**X**	**X**
Wavelet-HHH	firstorder	Median	—	—	—	—	X
Wavelet-HHH	firstorder	Uniformity	—	—	X	—	—
Wavelet-HHH	glcm	Cluster tendency	—	—	—	X	—
Wavelet-HHH	glcm	Inverse variance	X	—	—	—	—
Wavelet-HHH	glcm	Joint energy	—	X	—	—	—
Wavelet-HHH	glcm	MCC	X	—	X	—	—
Wavelet-HHH	glcm	Maximum probability	X	X	—	—	—
Wavelet-HHH	glcm	Sum entropy	X	X	X	X	—
Wavelet-HHH	gldm	Dependence entropy	X	X	—	X	X
Wavelet-HHH	glszm	Gray-level non-uniformity normalized	X	—	—	—	X
Wavelet-HHH	glszm	Gray-level variance	—	—	X	X	—
**Wavelet-HHH**	**glszm**	**Zone entropy**	**X**	**X**	**X**	**X**	**X**
Wavelet-HHH	ngtdm	Complexity	X	—	X	—	X
Wavelet-HHL	firstorder	Skewness	—	—	X	—	—
Wavelet-HHL	glcm	MCC	—	X	X	—	X
**Wavelet-HLH**	**firstorder**	**Skewness**	**X**	**X**	**X**	**X**	**X**
Wavelet-HLH	glcm	Cluster shade	—	—	X	—	—
Wavelet-HLH	gldm	Dependence entropy	X	—	—	—	X
Wavelet-HLH	glrlm	Long-run-high-gray-level emphasis	—	X	X	X	—
Wavelet-HLH	glrlm	Short-run-high-gray-level emphasis	—	X	—	—	—
Wavelet-HLH	glszm	Low-gray-level-zone emphasis	—	—	X	X	X
Wavelet-HLH	glszm	Small-area-high-gray-level emphasis	—	—	X	—	—
Wavelet-HLH	glszm	Small-area-low-gray-level emphasis	—	—	—	X	—
Wavelet-HLH	glszm	Zone entropy	—	—	—	—	X
Wavelet-HLL	glszm	Small-area-high-gray-level emphasis	—	—	—	—	X
Wavelet-LHH	firstorder	Median	—	X	—	—	—
Wavelet-LHH	glcm	Autocorrelation	—	X	—	—	—
Wavelet-LHH	glszm	Zone entropy	X	X	X	—	X
Wavelet-LHL	firstorder	Interquartile range	—	—	—	X	—
Wavelet-LHL	firstorder	Mean	—	X	X	X	—
Wavelet-LHL	firstorder	Median	—	—	—	X	—
Wavelet-LHL	firstorder	Skewness	—	X	—	—	—
Wavelet-LHL	glcm	Correlation	—	X	X	X	X
Wavelet-LHL	glrlm	Long-run-low-gray-level emphasis	—	—	—	X	—
Wavelet-LLH	glcm	Imc1	—	—	—	X	—
Wavelet-LLH	glcm	MCC	X	X	X	—	—
Wavelet-LLL	firstorder	10th percentile	X	—	—	—	—
Wavelet-LLL	firstorder	Median	—	—	—	X	—
Wavelet-LLL	glcm	Cluster shade	—	X	X	—	—
Wavelet-LLL	glcm	MCC	—	—	—	—	X
Wavelet-LLL	gldm	Large-dependence-high-gray-level emphasis	X	—	—	—	X
Wavelet-LLL	glrlm	Long-run-high-gray-level emphasis	—	X	—	—	—
Wavelet-LLL	glszm	Small-area-low-gray-level emphasis	—	—	—	X	—

**Fig. 6:**
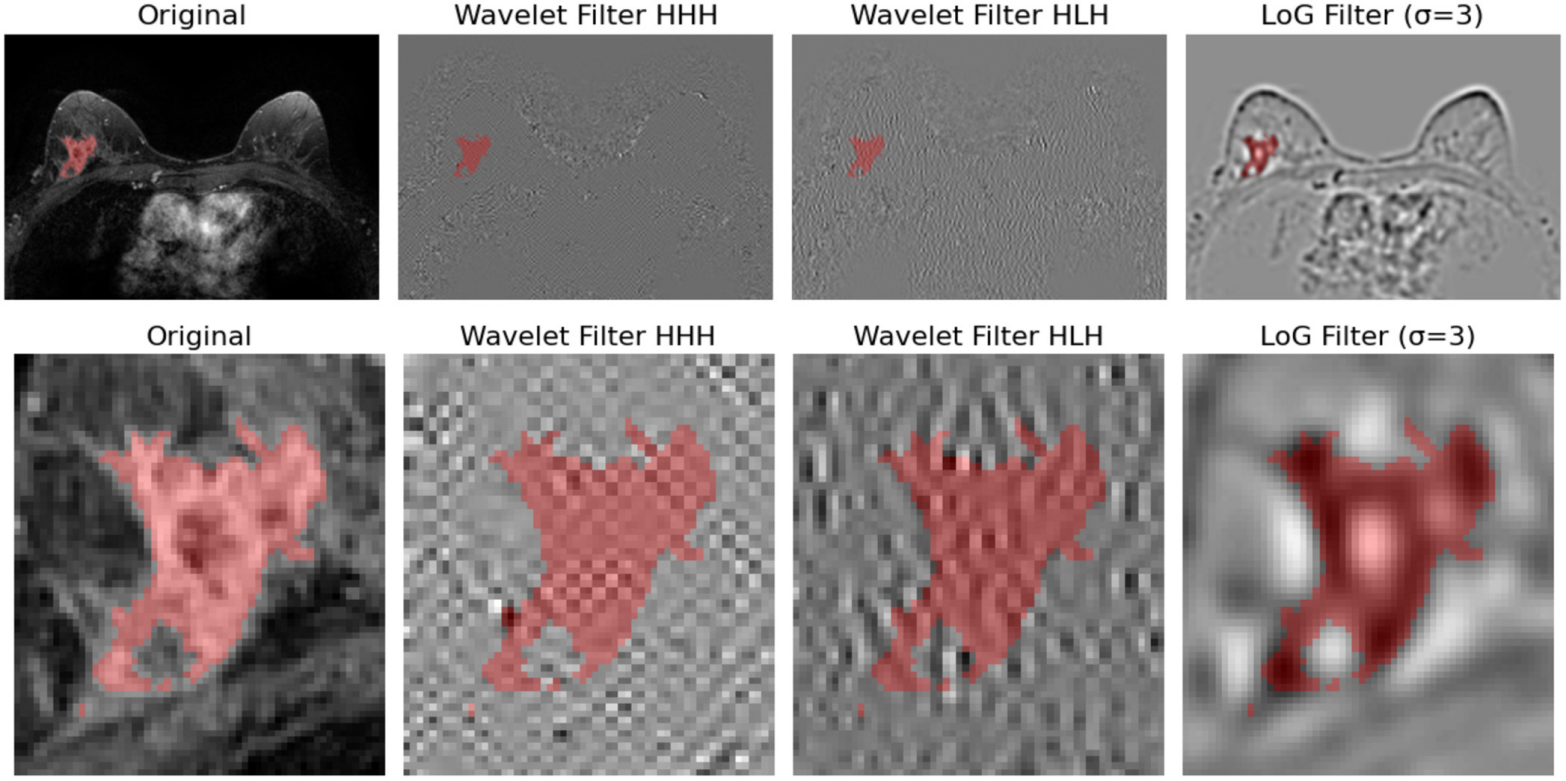
Top row, from left to right: example slice of original DCE-MR image, Wavelet-HHH filtered image, Wavelet-HLH filtered image, and LoG σ=3 filtered image. Bottom row, from left to right: zoomed view of the manual segmentation mask on the original and filtered images. Tumor segmentation is shown in red.

Only four of these features were commonly selected by the models and are reported in bold in Table 5: high-gray-level-zone emphasis (from LoG σ=3 image, GLSZM matrix), large-dependence high-gray-level emphasis (from original image and GLDM matrix), zone entropy (from wavelet-HHH image and GLSZM matrix), and skewness (from wavelet-HLH image, first-order features). Figure 6 reports an example of wavelet-HHH, wavelet-HLH, and LoG σ=3 filtered images, for a visual reference of the filtering effect.

**Fig. 7:**
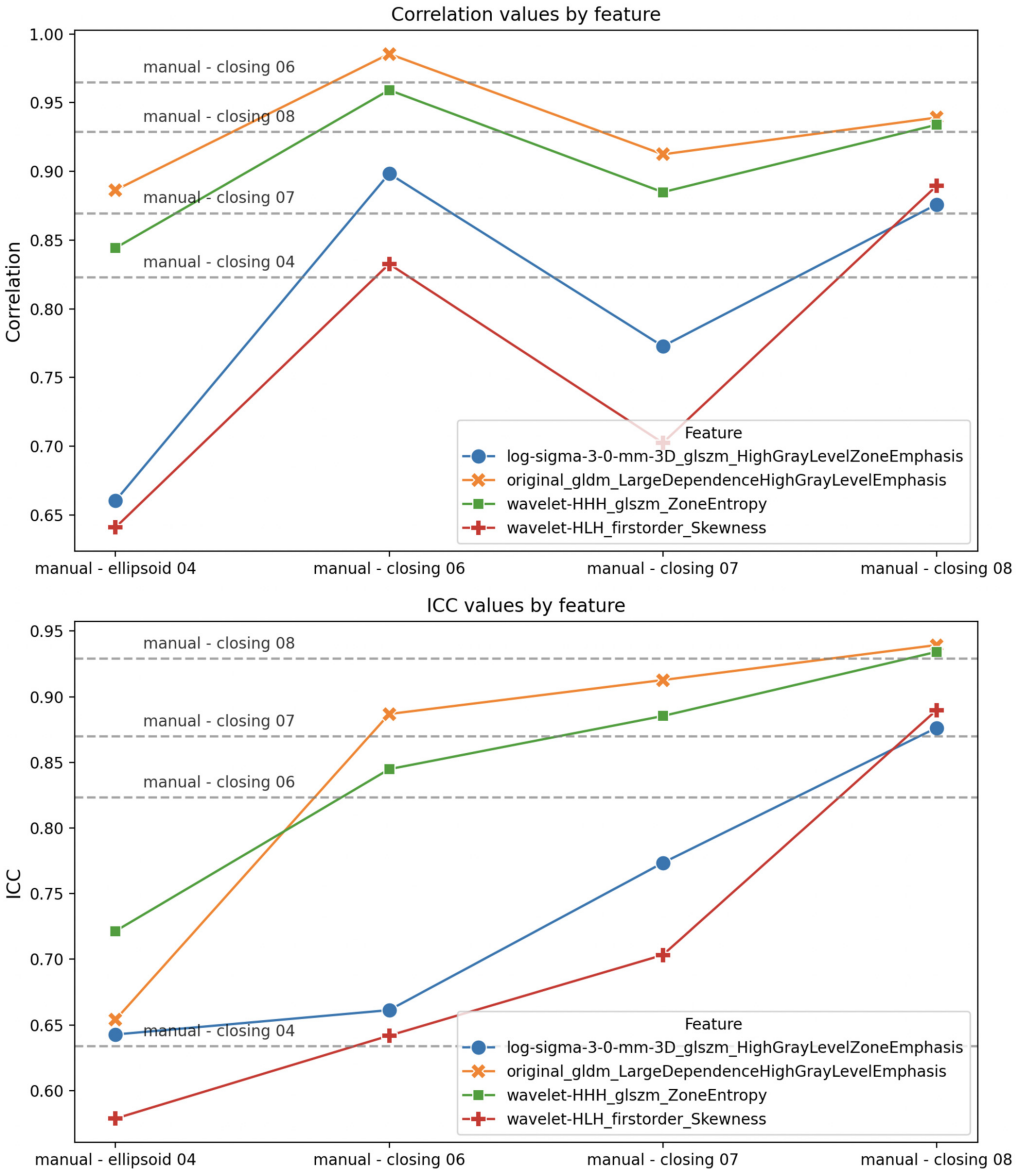
Top panel: ICC of the four common best-SHAP features at varying segmentation masks; the dashed lines indicate median ICC on all features, summarizing the reproducibility between features extracted from the manual mask and each of its over-segmentation modifications. Bottom panel: Pearson’s correlation of the four common features at varying masks; the dashed lines indicate median correlation on all features, summarizing the correlation between features extracted from the manual mask and each of its over-segmentation modifications.

For the stability analysis, we focused on the four commonly selected best-SHAP features cited above. Figure 7 displays the values of ICC and Pearson’s correlation for each of these features at varying segmentation masks (manual, closing 08, 07, 06, and ellipsoid 04). The dashed lines indicate the median ICC and Pearson’s correlation across all features at varying masks, for comparison with the scores of the four common features. These features exhibited decreasing ICC values as the segmentation accuracy declined (mean ICC across features for decreasing DSC: 0.910, 0.819, 0.759, 0.649) and a high Pearson’s correlation coefficient (mean Pearson’s correlation across features for decreasing DSC: 0.910, 0.818, 0.919, 0.758).

**Fig. 8:**
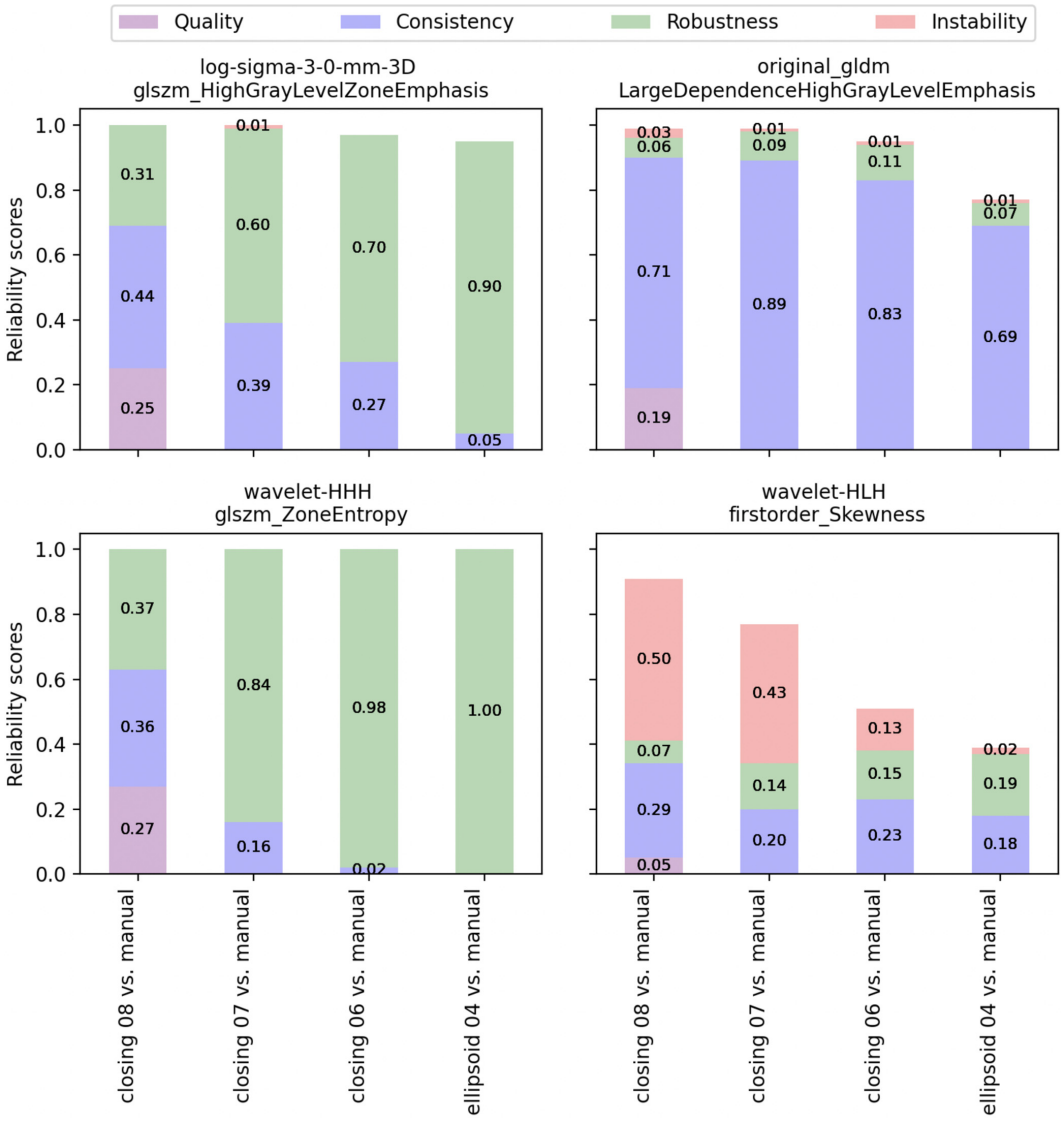
Reliability scores of the four common SHAP features, computed for each over-segmentation modification of the manual segmentation (manual versus closing 08, 07, 06, and ellipsoid 04).

**Fig. 9:**
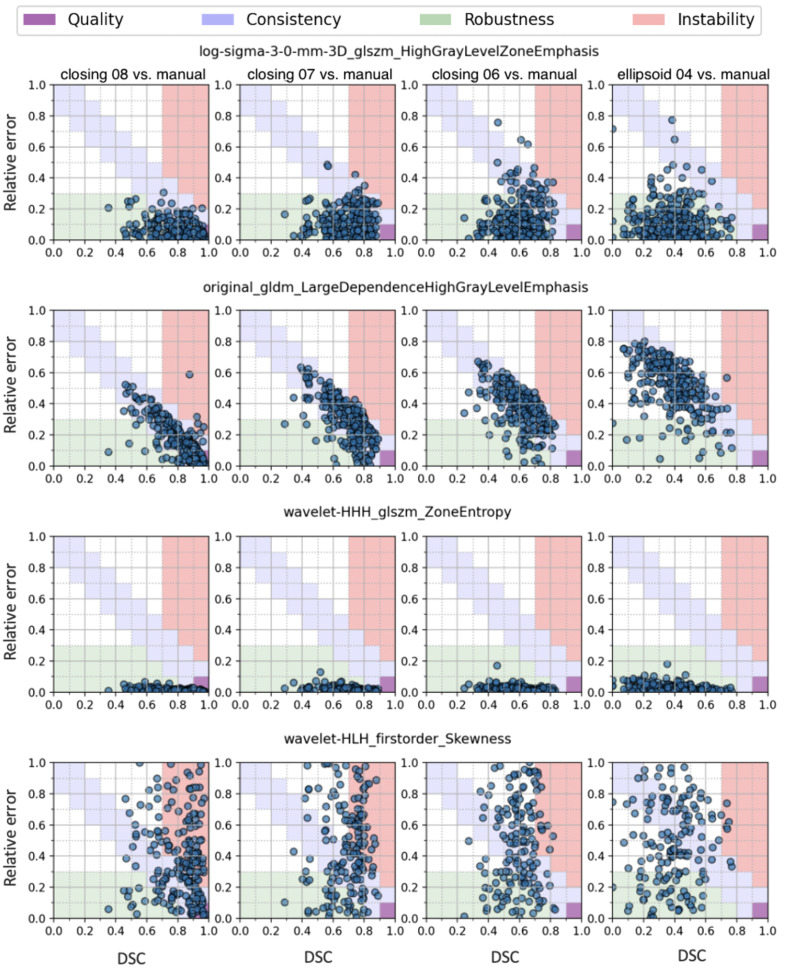
Scatter plots of relative error on feature value against DSC, for the four common SHAP features, across segmentation masks (from left to right: manual versus closing 08, 07, 06, and ellipsoid 04). The colored areas on the scatter plots correspond to the high-quality, consistency, robustness, and instability areas, as reported in the legend.

Figure 8 reports the reliability/stability scores of the four common SHAP features across mask modification, and Fig. 9 shows the scatter plots of feature stability with respect to segmentation variability for each of these features, for each over-segmentation modification of the manual mask. By definition, the scores may not sum to 1 if some patients fall outside the predefined ranges of quality, consistency, robustness, and instability. This is particularly evident for the wavelet-HLH-first order skewness feature (Fig. 9, last row), as decreasing accuracy of the segmentation mask leads to higher and higher relative error in the feature value for many patients (up to 60% of the patients, see bottom right panel of Fig. 8), causing them to fall outside the predefined ranges, and highlighting overall feature instability.

## 4 Discussion

We developed and evaluated ML models to investigate the role of radiomic features in distinguishing TNBC from other breast cancer subtypes (non-TNBC) across different segmentation masks. Two sets of radiomic features were used: an extended set obtained through an initial filtering step, and a refined subset comprising the most informative features identified by the SHAP method (best-SHAP features). In addition, we trained models using demographic and biopsy-derived clinical features for comparison.

Statistical tests show that no individual feature is able to differentiate TNBC and non-TNBC groups, which remarks the challenge of relying solely on radiomics for the automatic differentiation of breast cancer subtypes.

The results of the ML models show that using a selection of very explicative radiomic features (the best-SHAP features) leads to a prediction performance that is comparable to using biopsy variables, for the binary prediction of tumor subtypes.

We acknowledge that these biopsy variables are typically reported alongside other histopathological markers such as ER, PR, and HER2, which directly define TNBC subtype and are not intended for predictive modeling for TNBC. In the absence of other suitable variables for comparison with radiomics-based models, we employed biopsy-derived features associated with tumor aggressiveness (tubule formation, nuclear grade, and mitotic rate), which is in turn related to subtype. Although this connection is indirect, and the biopsy-based model does not constitute a true benchmark, we believe it provides a useful clinical point of reference for comparing imaging-based and nonimaging-based models.

Interestingly, prediction results of the best-SHAP models do not exhibit a significant statistical difference when compared with the best-SHAP manual model, i.e., models trained with features extracted from the manual segmentation and its variations produce comparable performance (see Fig. 4 and Table 4). The prediction obtained for the ellipsoid 04 mask (an ellipsoid over the region of interest provided by the dataset) is particularly surprising. Not only this segmentation mask is extremely poor in terms of DSC, but it also represents a very rough approximation of the tumor shape, missing all contour details and incorporating contrast and irregularity of the surrounding regions of the tumor, such as veins, necrosis, edema, and fat tissue (see Fig. 1, bottom row). The results obtained with the opening masks are slightly lower on average (see Table 3, best-SHAP opening models), and the statistical tests show that including partial peritumoral information, through over-segmentation masks, contributes to a better predictive performance than the one obtained from the under-segmentation masks (see Table 4). This fact is also supported by literature as various papers showed the contribution of peritumoral information for predicting TNBC subtype or response to neoadjuvant therapy in TNBC patients.^40^^–^^42^ Moreover, Fig. 5 shows that the aggregated performances of the closing-ellipsoid models, obtained through over-segmentation, are overall better (p<0.05) than the ones of the opening models, obtained through under-segmentation. In addition, to address the heterogeneity and randomness typical of inter-observer variability in clinical practice, we introduced random segmentation variability, as described in Sec. 2.2. The predictive performances obtained by randomizing manual and over-segmentation masks, manual and under-segmentation, or all the possible segmentation modifications resulted to be comparable to the performances of the other best-SHAP models, including the manual one (see Fig. 4 and Table 4). The only exception is provided by the random manual-opening model, showing a statistical difference from the biopsy-based model and two over-segmentation models (best-SHAP closing 07 and 06), coherently with the result reported in Fig. 5 and discussed above. This analysis underscores that random variability in segmentation has a limited impact on the predictive result.

In summary, a systematic overestimation of the segmentation mask yields better predictive performance than a systematic underestimation, whereas heterogeneous segmentation error has a limited impact on model performance.

These results motivated the need to investigate the best-SHAP features selected by the models. We concentrated the stability analysis on the features obtained from manual and over-segmentation models (manual, closing 08, 07, 06, and ellipsoid 04), focusing on the features that were commonly selected by these five models as they could elucidate behaviors regarding their stability and robustness across segmentation masks and offer insights into the factors influencing the prediction results. Figure 7 compares the ICC and Pearson’s correlation of each common feature, both computed between features extracted from the manual mask and its variations (closing 08, 07, 06, and ellipsoid 04). As a general trend, the curves associated with original-gldm large-dependence high-gray-level emphasis and wavelet-HHH-glszm zone entropy (orange and green) are above the ones associated to than LoG σ=3-glszm High-gray-level-zone emphasis and wavelet-HLH-first order skewness (blue and red), both for ICC and correlation coefficient. We can notice that, according to thresholds reported in literature,^16^^,^^43^ LoG σ=3-glszm high-gray-level-zone emphasis and wavelet-HLH-first order skewness would have been discarded by the feature selection step (ICC < 0.9) from the prediction model based on the manual mask. However, despite their low ICC values (e.g., lower than 0.75), these features are still considered important and are consistently selected in all predictive models. This observation suggests that relying solely on ICC for feature selection may not be the most effective approach. Specifically, when examining the correlation coefficient, we find cases where a low ICC does not necessarily correspond to a low correlation coefficient, see Fig. 7. This discrepancy arises because the ICC is penalized when the inter-rater variance is high (raters are represented by segmentation masks), whereas the correlation coefficient ignores inter-rater effects and merely measures the correlation of feature values across different segmentations. The high correlation of a predictive feature with its corresponding values extracted from another segmentation mask suggests that both these features are capturing meaningful information relevant to the target, despite the poor reliability rated by ICC.

In addition, the computation of the reliability scores for the common features, in Fig. 8, highlights that there is no shared numerical relationship between feature stability and segmentation variability among the predictive features. However, segmentation variability does not disrupt the feature stability pattern of individual features, as robust, consistent, and unstable patterns remain well-defined across mask modifications, as displayed in Fig. 9. Moreover, from Fig. 9, it emerges that three of our most discriminative/predictive features (i.e., original-gldm large-dependence high-gray-level emphasis, LoG σ=3-glszm high-gray-level-zone emphasis, and wavelet-HLH-first order skewness) are indeed dependent on the segmentation process and are not invariant with respect to segmentation. Nevertheless, these features are consistently selected as predictive, and importantly, the predictive performances remain comparable although these features are not stable under segmentation variability.

These considerations on the presented results suggest that the feature selection process in predictive models is not inherently linked to feature stability with respect to segmentation variability, neither from a numerical nor from a reproducibility perspective (ICC), but rather to general patterns captured by the distribution of features across patients. This underscores why feature selection approaches based on ICC or reliability scores may fail to identify the most predictive features for a specific task. On the other hand, the high correlation between the same predictive features extracted from different segmentation masks helps explain why the best-SHAP models yield similar performance.

Overall, the results presented here suggest that high segmentation accuracy may not be a necessary requirement for radiomics applications and that feature stability with respect to segmentation variability is not a strict prerequisite for predictive relevance.

This study presents some limitations. First, our findings are context-specific (TNBC prediction using the Duke dataset on the first post-contrast MRI) and do not imply that segmentation precision is not important for all radiomic tasks. Indeed, the impact of segmentation dependency on predictive performance can vary depending on the specific context and application. Second, the variability range of the ROC-AUC is not particularly narrow, a concern previously highlighted by del Angel et al.^44^ in other radiomics-based applications for breast cancer. Third, the dataset we used was obtained from a single institution; therefore, no independent testing was conducted. Although cross-validation helps reduce overfitting on specific test sets, future work should involve validating the method with larger, multi-institutional datasets to better assess reproducibility and generalizability of the results. Indeed, it is worth noting that differences in the segmentations can lead to the selection of different features, which may impact model interpretability. However, despite this variability, in this work, we identified a subset of features that are consistently predictive across segmentation variations for our TNBC versus non-TNBC task, and we observe that the model performance, in terms of ROC-AUC, remains stable across segmentation variability (from 0.8 DSC to 0.4 DSC with respect to manual segmentation). This finding supports the robustness and generalizability of the proposed pipeline, providing a basis for further validation in multicenter settings.

In addition, the set of extracted first-post-contrast texture features and tumor morphology features may not adequately capture the full spectrum of complexities involved in tumor subtype classification. Future investigations should incorporate time-dependent texture descriptors and heterogeneity features related to enhancement kinetics into the ML models, as illustrated by Caballo et al.^31^ for other prediction purposes. Future work may also include features extracted from other MRI time points, MRI sequences, e.g., diffusion weighted imaging, and ADC, to further improve cancer characterization.

## 5 Conclusion

This study examined the performance of radiomics-based ML models for distinguishing TNBC from other subtypes across varying segmentation masks, generated through controlled modifications of manual breast lesion segmentations. Our findings indicate that TNBC prediction performance remains largely unaffected by segmentation variability. This suggests that highly precise segmentation may not be a strict requirement for radiomics applications and that including partial peritumoral regions does not necessarily compromise the predictive value of radiomic features. Furthermore, analysis of reproducibility metrics and reliability scores for the most predictive features highlights a potential drawback of relying exclusively on feature selection methods based on stability across segmentations. Such approaches may inadvertently discard informative features that contribute to predictive performance. It is plausible that if radiomic features had greater intrinsic predictive power, segmentation variability would exert a more noticeable effect on model accuracy. However, radiomics-based predictions remain modest–partly due to limited sample size, but primarily because ML models struggle to identify consistently predictive features across random data splits without overfitting. This limitation reinforces the need for robust, explainable, and population-level feature selection strategies.

By shedding light on the complex relationship between feature stability and predictive result, this work challenges the assumption that precise segmentation is always critical for feature selection and predictive performance. It opens the possibility that, in some scenarios, particularly predictive modeling, radiomics may tolerate greater segmentation variability than expected, without sacrificing outcome quality. Ultimately, this work supports the development of more flexible and time-efficient radiomics pipelines, potentially enhancing their feasibility and scalability for clinical integration.

## Data Availability

Data used for the analysis can be downloaded from Synapse at https://doi.org/10.7303/syn60868042 as part of the Duke dataset.^29^^,^^45^ The exact list of images and the codes used for the analysis are available upon reasonable request.

## References

[1] Johnson K. S. Conant E. F. Soo M. S., “Molecular subtypes of breast cancer: a review for breast radiologists,” J. Breast Imaging 3(1), 12–24 (2021). 10.1093/jbi/wbaa110 38424845

[2] Foulkes W. D. Smith I. E. Reis-Filho J. S., “Triple-negative breast cancer,” N. Engl. J. Med. 363(20), 1938–1948 (2010). NEJMAG 0028-4793 10.1056/NEJMra1001389 21067385

[3] Bianchini G. et al., “Triple-negative breast cancer: challenges and opportunities of a heterogeneous disease,” Nat. Rev. Clin. Oncol. 13(11), 674–690 (2016). 10.1038/nrclinonc.2016.66 27184417 PMC5461122

[4] Sha Y. Chen J., “MRI-based radiomics for the diagnosis of triple-negative breast cancer: a meta-analysis,” Clin. Radiol. 77(9), 655–663 (2022). 10.1016/j.crad.2022.04.015 35641339

[5] Gillies R. J. Kinahan P. E. Hricak H., “Radiomics: images are more than pictures, they are data,” Radiology 278(2), 563–577 (2016). RADLAX 0033-8419 10.1148/radiol.2015151169 26579733 PMC4734157

[6] Zwanenburg A. et al., “The image biomarker standardization initiative: standardized quantitative radiomics for high-throughput image-based phenotyping,” Radiology 295(2), 328–338 (2020). RADLAX 0033-8419 10.1148/radiol.2020191145 32154773 PMC7193906

[7] Rizzo S. et al., “Radiomics: the facts and the challenges of image analysis,” Eur. Radiol. Exp. 2(1) (2018). 10.1186/s41747-018-0068-z PMC6234198 30426318

[8] Conti A. et al., “Radiomics in breast cancer classification and prediction,” Semin. Cancer Biol. 72, 238–250 (2021). 10.1016/j.semcancer.2020.04.002 32371013

[9] Tagliafico A. S. et al., “Overview of radiomics in breast cancer diagnosis and prognostication,” Breast 49, 74–80 (2020). 10.1016/j.breast.2019.10.018 31739125 PMC7375670

[10] Son J. et al., “Prediction of breast cancer molecular subtypes using radiomics signatures of synthetic mammography from digital breast tomosynthesis,” Sci. Rep. 10(1), 21566 (2020). SRCEC3 2045-2322 10.1038/s41598-020-78681-9 33299040 PMC7726048

[11] Zou H. Hastie T., “Regularization and variable selection via the elastic net,” J. R. Stat. Soc. Ser. B Stat. Methodol. 67, 301–320 (2005). 10.1111/j.1467-9868.2005.00503.x

[12] Leithner D. et al., “Non-invasive assessment of breast cancer molecular subtypes with multiparametric magnetic resonance imaging radiomics,” J. Clin. Med. 9(6), 1853 (2020). 10.3390/jcm9061853 32545851 PMC7356091

[13] Cama I. et al., “A study on the role of radiomics feature stability in predicting breast cancer subtypes,” Proc. SPIE 13174, 131741O (2024). 10.1117/12.3027015

[14] Cama I. et al., “Segmentation agreement and the reliability of radiomics features,” Adv. Comput. Sci. Eng. 1(2), 202–217 (2023). 10.3934/acse.2023009

[15] Scalco E. Rizzo G. Mastropietro A., “The stability of oncologic MRI radiomic features and the potential role of deep learning: a review,” Phys. Med. Biol. 67, 09TR03 (2022). 10.1088/1361-6560/ac60b9 35325881

[16] Granzier R. W. Y. et al., “MRI-based radiomics in breast cancer: feature robustness with respect to inter-observer segmentation variability,” Sci. Rep. 10(1), 14163 (2020). SRCEC3 2045-2322 10.1038/s41598-020-70940-z 32843663 PMC7447771

[17] Radiomics.bio, “RadioMix Toolbox,” https://radiomics.bio/radiomix-toolbox/ (2021).

[18] van Griethuysen J. J. M. et al., “Computational radiomics system to decode the radiographic phenotype,” Cancer Res. 77(21), e104–e107 (2017). CNREA8 0008-5472 10.1158/0008-5472.CAN-17-0339 29092951 PMC5672828

[19] Koo T. K. Li M. Y., “A guideline of selecting and reporting intraclass correlation coefficients for reliability research,” J. Chiropr. Med. 15, 155–163 (2016). 10.1016/j.jcm.2016.02.012 27330520 PMC4913118

[20] Pistel M. et al., “Stability of radiomic features against variations in lesion segmentations computed on apparent diffusion coefficient maps of breast lesions,” Diagnostics 14(13), 1427 (2024). 10.3390/diagnostics14131427 39001317 PMC11241112

[21] Ponsiglione A. M. et al., “A statistical approach to assess the robustness of radiomics features in the discrimination of mammographic lesions,” J. Pers. Med. 13(7), 1104 (2023). 10.3390/jpm13071104 37511717 PMC10381882

[22] Zwanenburg A. et al., “Assessing robustness of radiomic features by image perturbation,” Sci. Rep. 9(1), 614 (2019). SRCEC3 2045-2322 10.1038/s41598-018-36938-4 30679599 PMC6345842

[r23] Yang F. et al., “Impact of contouring variability on oncological PET radiomics features in the lung,” Sci. Rep. 10(1), 369 (2020). SRCEC3 2045-2322 10.1038/s41598-019-57171-7 31941949 PMC6962150

[24] Tixier F. et al., “Reliability of tumor segmentation in glioblastoma: impact on the robustness of MRI-radiomic features,” Med. Phys. 46(8), 3582–3591 (2019). MPHYA6 0094-2405 10.1002/mp.13624 31131906 PMC6692188

[25] Kothari G. et al., “The impact of inter-observer variation in delineation on robustness of radiomics features in non-small cell lung cancer,” Sci. Rep. 12(1), 12822 (2022). SRCEC3 2045-2322 10.1038/s41598-022-16520-9 35896707 PMC9329346

[26] Poirot M. G. et al., “Robustness of radiomics to variations in segmentation methods in multimodal brain MRI,” Sci. Rep. 12(1), 16712 (2022). SRCEC3 2045-2322 10.1038/s41598-022-20703-9 36202934 PMC9537186

[27] Liu R. et al., “Stability analysis of CT radiomic features with respect to segmentation variation in oropharyngeal cancer,” Clin. Transl. Radiat. Oncol. 21, 11–18 (2020). 10.1016/j.ctro.2019.11.005 31886423 PMC6920497

[28] Hatamikia S. et al., “Breast MRI radiomics and machine learning-based predictions of response to neoadjuvant chemotherapy—how are they affected by variations in tumor delineation?,” Comput. Struct. Biotechnol. J. 23, 52–63 (2024). 10.1016/j.csbj.2023.11.016 38125296 PMC10730996

[29] Saha A. et al., “Dynamic contrast-enhanced magnetic resonance images of breast cancer patients with tumor locations [Data set],” The Cancer Imaging Archive, 10.7937/TCIA.e3sv-re93 (2021).

[30] Clark K. et al., “The cancer imaging archive (TCIA): maintaining and operating a public information repository,” J. Digit. Imaging 26(6), 1045–1057 (2013). JDIMEW 10.1007/s10278-013-9622-7 23884657 PMC3824915

[31] Caballo M. et al., “Four-dimensional machine learning radiomics for the pretreatment assessment of breast cancer pathologic complete response to neoadjuvant chemotherapy in dynamic contrast-enhanced MRI,” J. Magn. Reson. Imaging 57(1), 97–110 (2023). 10.1002/jmri.28273 35633290 PMC10083908

[32] Van der Walt S. et al., “scikit-image: image processing in Python,” PeerJ 2, e453 (2014). 10.7717/peerj.453 25024921 PMC4081273

[33] Zhou J. et al., “Predicting the response to neoadjuvant chemotherapy for breast cancer: wavelet transforming radiomics in MRI,” BMC Cancer 20(1), 100 (2020). BCMACL 1471-2407 10.1186/s12885-020-6523-2 32024483 PMC7003343

[34] Demircioğlu A., “The effect of preprocessing filters on predictive performance in radiomics,” Eur. Radiol. Exp. 6(1), 40 (2022). 10.1186/s41747-022-00294-w 36045274 PMC9433552

[35] Orlhac F. et al., “A guide to ComBat harmonization of imaging biomarkers in multicenter studies,” J. Nucl. Med. 63(2), 172–179 (2022). JNMEAQ 0161-5505 10.2967/jnumed.121.262464 34531263 PMC8805779

[36] Whitney H. M. et al., “Harmonization of radiomic features of breast lesions across international DCE-MRI datasets,” J. Med. Imaging 7(1), 012707 (2020). JMEIET 0920-5497 10.1117/1.JMI.7.1.012707 PMC7056633 32206682

[37] Chawla N. V. et al., “SMOTE: synthetic minority over-sampling technique,” J. Artif. Intell. Res. 16, 321–357 (2002). JAIRFR 1076-9757 10.1613/jair.953

[38] Nick T. G. Campbell K. M., Logistic Regression, Humana Press, Totowa, New Jersey, pp. 273–301 (2007). 10.1007/978-1-59745-530-5_14 18450055

[39] Lundberg S. M. Lee S.-I., “A unified approach to interpreting model predictions,” in Adv. Neural Inf. Process. Syst. 30 (2017).

[40] Panthi B. et al., “Longitudinal dynamic contrast-enhanced MRI radiomic models for early prediction of response to neoadjuvant systemic therapy in triple-negative breast cancer,” Front. Oncol. 13, 1264259 (2023). FRTOA7 0071-9676 10.3389/fonc.2023.1264259 37941561 PMC10628525

[r41] Niu S. et al., “Intra- and peritumoral radiomics on assessment of breast cancer molecular subtypes based on mammography and MRI,” J. Cancer Res. Clin. Oncol. 148(1), 97–106 (2022). JCROD7 1432-1335 10.1007/s00432-021-03822-0 34623517 PMC11800953

[42] Han Y. et al., “The value of intratumoral and peritumoral radiomics features based on multiparametric MRI for predicting molecular staging of breast cancer,” Front. Oncol. 15, 1379048 (2025). FRTOA7 0071-9676 10.3389/fonc.2025.1379048 40134601 PMC11933106

[43] Caballo M. et al., “Multi-marker quantitative radiomics for mass characterization in dedicated breast CT imaging,” Med. Phys. 48(1), 313–328 (2021). MPHYA6 0094-2405 10.1002/mp.14610 33232521 PMC7898616

[44] del Angel R. M. et al., “Added value of feature uncertainty in a radiomic analysis of contrast-enhanced digital mammography boosted by deep learning,” Proc. SPIE 13174, 1317403 (2024). PSISDG 0277-786X 10.1117/12.3027034

[45] Garrucho L. et al., “A large-scale multicenter breast cancer DCE-MRI benchmark dataset with expert segmentations,” Sci. Data 12(1), 453 (2025). 10.1038/s41597-025-04707-4 40108146 PMC11923173

